# Laparoscopic vs. Open Surgical Repair of Subxiphoidal Hernia Following Median Sternotomy for Coronary Bypass - Analysis of the Herniamed Registry

**DOI:** 10.3389/fsurg.2020.580116

**Published:** 2020-11-09

**Authors:** Hendrik C. Albrecht, Mateusz Trawa, Ferdinand Köckerling, Martin Hukauf, Stephan Gretschel

**Affiliations:** ^1^Department of General, Visceral, and Thoracic Surgery, Faculty of Health Sciences Brandenburg, Brandenburg Medical School Theodor Fontane, University Hospital Neuruppin, Neuruppin, Germany; ^2^Department of Surgery, Center for Minimally Invasive Surgery, Academic Teaching Hospital of Charité Medical School, Vivantes Hospital, Berlin, Germany; ^3^StatConsult GmbH, Magdeburg, Germany

**Keywords:** subxiphoidal, incisional hernia, recurrence, outcome, laparoscopic, open repair

## Abstract

**Introduction:** The repair of subxiphoidal incisional hernia following median sternotomy is technically demanding due to the specific anatomic situation and the lateral distracting forces in this region. Published data are available from retrospective reports with limited number of patients only. The aim of this study was to evaluate the outcome of subxiphoidal hernia repair comparing laparoscopic and open surgical approach.

**Materials and Methods:** This analysis of Herniamed registry data of patients with subxiphoidal incisional hernia following sternotomy for coronary bypass assesses the perioperative and 1 year follow-up outcome of laparoscopic and open repair. Demographic data and perioperative outcomes were stratified by surgical approach (laparoscopic vs. open) and compared as unadjusted analyses using Chi square and Students *t*-tests.

**Results:** Of 208 patients identified for the analysis 69 patients (33.2%) underwent laparoscopic and 139 (66.8%) patients had open repair. Concerning demographic data (gender, age, BMI, ASA score), risk factors and hernia size there were no significant differences between laparoscopic and open repair group. For intraoperative, postoperative and general complications as well as complication related re-operations no significant differences were seen between the groups. No significant advantage could be stated for laparoscopic repair regarding duration of operation and hospital stay. The recurrence rate at 1 year follow-up was higher in the laparoscopic group (7.2 vs. 2.2%; *p* = 0.072). No significant differences were reported in the 1 year follow-up evaluation of pain at rest, pain on exertion and pain requiring treatment.

**Conclusion:** The repair of subxiphoidal incisional hernia is safe in both open and laparoscopic technique. With regard to the lower recurrence rate preference can be given to open repair.

## Introduction

Subxiphoidal incisional hernia is a complication following median sternotomy with a reported incidence up to 4.2% (1, 2). The actual hernia rate might be underestimated as small hernia remain asymptomatic and the left lobe of the liver often prevents symptomatic incarceration (3). Long term data from a large cohort of patients are missing.

Reported predisposing factors for subxiphoidal incisional hernia are male sex, obesity, postoperative wound infection, and left ventricular failure (1, 4).

**Figure 1:**
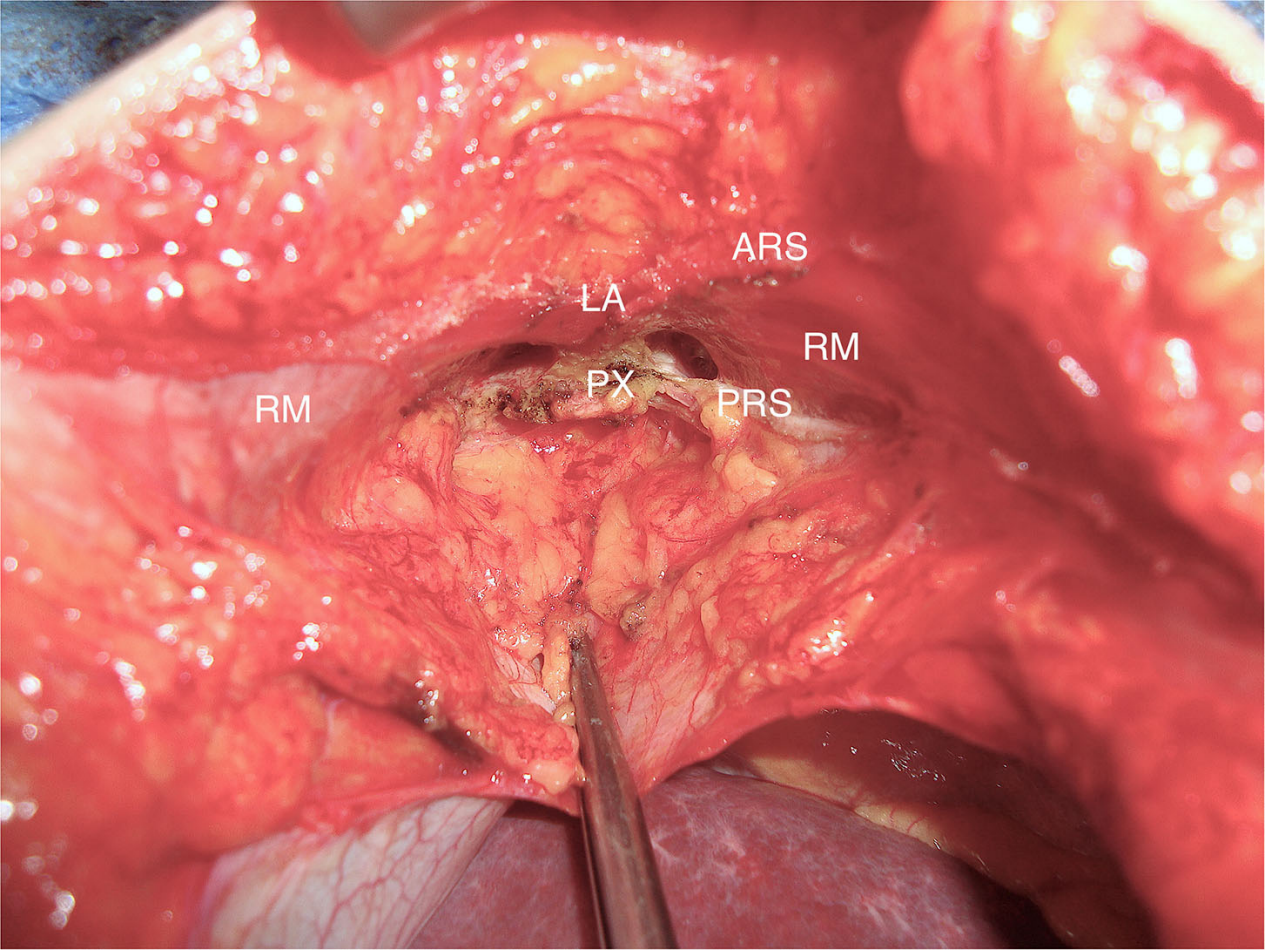
Subxiphoidal situs. *RM*, rectus muscle; PX, processus xiphoideus; LA, linea alba; ARS, anteroir lamina of rectus sheath; PRS, posterior lamina of rectus sheath.

In the subxiphoidal region fascia defects are closely located to cartilaginous and osseous structures of the rib cage and xiphoid and to the sternal potion of the diaphragm (Figure 1).

The repair of subxiphoidal hernia is rather demanding due to the special anatomic situation and the lateral distracting forces during respiration and coughing (2, 5).

Different techniques have been introduced for open surgical and laparoscopic hernia repair (3, 5–11). Limited data from retrospective studies give only restricted orientation concerning the preferred repair of subxiphoidal hernia.

The aim of this study was to evaluate the perioperative and the 1-year follow up outcome of laparoscopic vs. open surgical repair based on the analysis of data of the Herniamed Registry.

## Patients and Methods

The Herniamed Registry is an internet based multicenter quality assurance study that started in January 2009 (12, 13). Participating hospitals (737 in February 2020) and surgeons in private practice in Germany, Austria, and Switzerland prospectively enter patients data of routine hernia surgery to the registry. Patients signed informed consent concerning the registry and the obligation to inform the surgeon in case of any problems following the operation (14). All postoperative complications within 30 days are documented.

In a 1-year follow up questionnaire both general practitioner and patient are asked about recurrence, pain at rest or exertion, chronic pain requiring treatment and postoperative complications are reviewed once again. In case of reported recurrence or chronic pain patients are requested to attend for clinical examination and radiological tests.

The surgical technique followed general principles of tension-free incisional hernia repair. In most cases, non-absorbable mesh prostheses with an overlap of at least 5 cm were used. In laparoscopic repair, the mesh prothesis is placed in intraperitoneal onlay position. The falciform ligament is detached to ensure adequate cranial mesh overlap up to the liver veins and esophagus. The closure of the hernia defect is not obligatory. The mesh fixation in laparoscopic technique is performed with anchoring sutures and/or endoscopic tacks. Various methods were applied for the open repair. In standard sublay repair, the posterior lamina of the rectus sheath is separated from the xiphoid process to open the retroxiphoid space and provide a sufficient cranial mesh underlay (Figure 1). After closure of the peritoneum and the posterior lamina of the rectus sheath, the mesh prosthesis is placed in retromuscular and retroxiphoid position. The tension-free closure of the anterior lamina of the rectus sheath is performed for midline reconstruction.

In the following analysis data of patients with subxiphoidal incisional hernia following sternotomy for coronary bypass were analyzed to compare perioperative and 1 year follow-up outcome for laparoscopic vs. open surgical repair. Patients inclusion criteria were minimum age of 16 years, classification of subxiphoidal hernia only (no combinations), operation date before January 2019, previous operation coronary bypass and availability of 1 year follow-up data.

Hernia size was categorized according to European Hernia Society (EHS) classification: W1 < 4 cm, W2 ≥ 4–10 cm, W3 > 10 cm (15).

Besides demographic and patient related parameters, predisposing factors, intra- and postoperative complications, recurrence rates and pain rates were analyzed.

Data were stratified by surgical approach (laparoscopic vs. open) and as unadjusted analyses, differences between procedures were assessed using the asymptotic Chi-Square test for categorical parameters and the robust *t*-test (Satterthwaite) for continuous parameters, respectively.

Furthermore, since the analysis population was restricted to patients with 1-year follow-up, standardized differences were estimated to quantify differences in distribution between patients with and without follow-up. As a rule of thumb, a good balance between the groups and thus comparability is assured by a standardized difference of <10%. But for very small sample sizes, this amount is easily exceeded.

All analyses were performed using SAS 9.4 – software (SAS Institute Inc., Cary, NC, USA) and intentionally calculated to a full significance level of 5%, no corrections were made for multiple testing, each *p-*value ≤ 0.05 represents a significant result.

## Results

**Figure 2:**
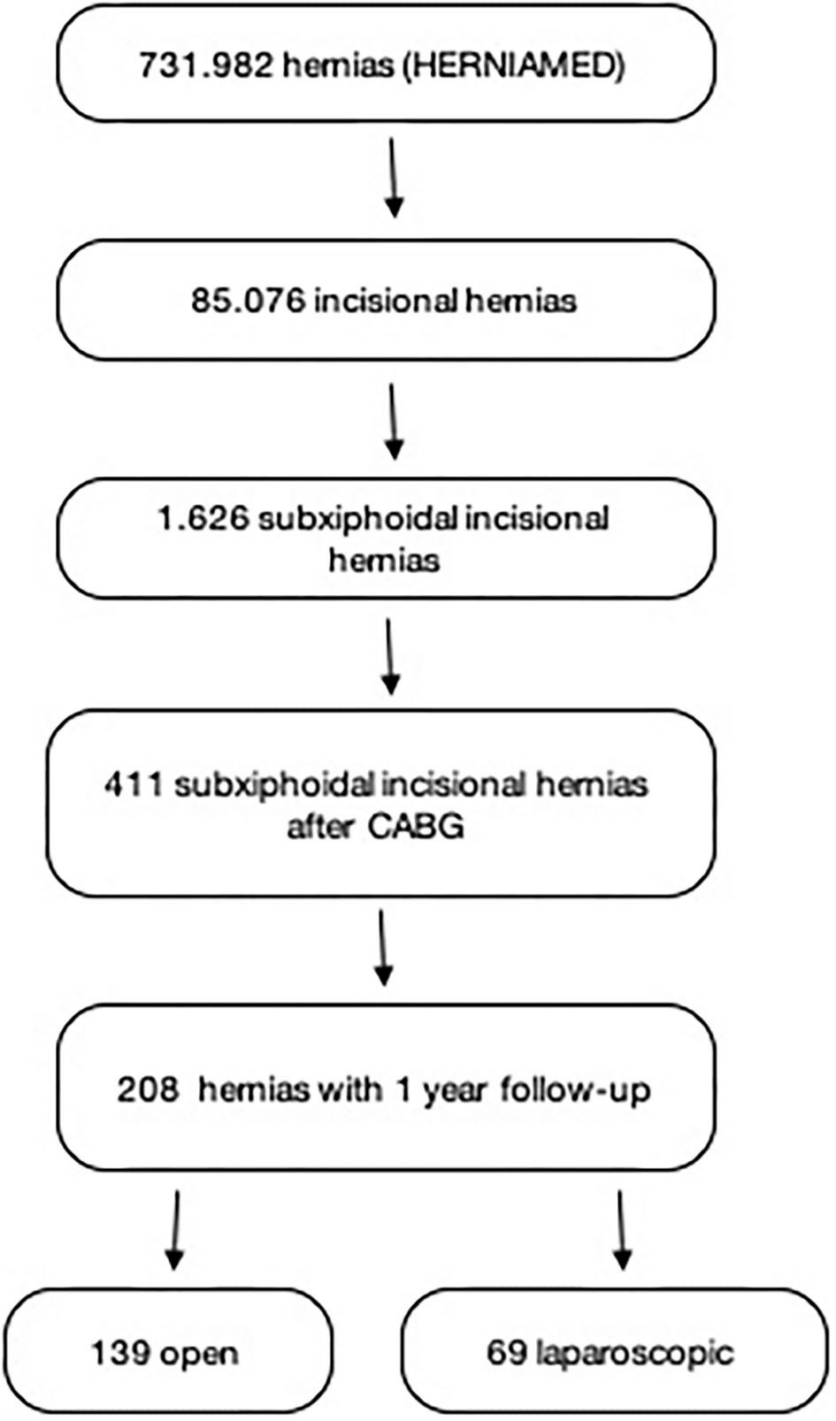
Flowchart of patient inclusion.

According to selection criteria 208 patients were identified for analysis out of 731.982 patients in the registry (Figure 2). Among these patients 12 were treated for recurrent hernia. Sixty-nine patients (33.2%) underwent hernia repair in laparoscopic technique (lap), 139 patients (66.8%) had open surgical repair (open). Of the latter 92 patients (44.2%) received sublay repair, 22 patients (10.6%) had open IPOM, 10 patients (4.8%) were operated in onlay technique. Further 14 patients (6.7%) in the open repair group underwent conventional primary midline approximation and suture repair of the fascia, one patient (0.5%) had component separation. All laparoscopic operations were performed in IPOM-technique (Table 1).

**Table 1:** Operation technique.

**Procedure**	***n* (%)**
Laparoscopic (IPOM)	69 (33.17)
Open	139 (66.83)
Sublay	92 (44.23)
IPOM	22 (10.58)
Direct suture	14 (6.73)
Onlay	10 (4.81)
Component separation	1 (0.48)

Concerning demographic data as gender, age, BMI, ASA score and risk factors no significant differences were found in unadjusted analyses between the patients that received laparoscopic and those with open surgical repair. The distribution of hernia defect size according to EHS classification did not differ between the groups lap vs. open. Furthermore, there was no significant difference concerning the number of patients treated for recurrent hernia between the groups (Table 2).

**Table 2:** Demographic data, risk factors, hernia size.

	**Procedure**			
	**Open**	**Laparoscopic**	***p***	**All patients**
	***n* (%)**	***n* (%)**		***n* (%)**
**Gender**				
Male	117 (84.2)	65 (94.2)	0.04	182 (87.5)
Female	22 (15.8)	4 (5.8)		26 (12.5)
**BMI**				
Underweight	1 (0.7)	0 (0)	0.84	1 (0.5)
Normal weight	37 (26.8)	16 (23.2)		53 (25.5)
Overweight	51 (37.0)	27 (39.1)		78 (37.5)
Obesity/morbid	49 (35.5)	26 (37.7)		75 (36.1)
**ASA score**				
I	0 (0)	1 (1.4)	0.22	1 (0.5)
II	42 (30.2)	16 (23.2)		58 (27.9)
III/IV	97 (69.8)	52 (75.4)		149 (71.6)
**Risk factors**				
COPD	18 (12.9)	10(14.5)	0.76	28 (13.5)
Diabetes	22 (15.8)	7 (10.1)	0.27	29 (13.9)
Smoking	12 (8.6)	9(13.0)	0.32	21 (10.1)
Coagulopathy	6 (4.3)	1 (1.4)	0.28	7 (3.4)
Aortic aneurysm	4 (2.9)	3 (4.3)	0.58	7 (3.4)
Immunosupression	1 (0.7)	1 (1.4)	0.61	2 (1.0)
**Defect size (incisional)**				
WI (<4 cm)	72 (51.8)	30 (43.5)	0.46	102 (49.0)
WII (4–10 cm)	64 (46.0)	38 (55.1)		102 (49.0)
WIII (>10 cm)	3 (2.2)	1 (1.4)		4 (1.9)
Recurrent operation	9 (6.5)	3 (4.3)	0.54	12 (5.7)

With regard to technical aspects - closure of the hernia defect was performed less frequently in laparoscopic hernia repair (23.6 vs. 45.8%; *p* = 0.005). The mean size of the used mesh prothesis was bigger in laparoscopic repair 228.4 (226.6–230.1) cm^2^ vs. 105.5 (103.0–107.5) cm^2^ (*p* < 0.001). The rate of mesh fixation was higher in the laparoscopic group (98.6 vs. 74.1%; *p* < 0.001).

No significant differences between the groups were seen for intraoperative, general and postoperative complications, complication related re-operations and mean length of hospital stay. The mean duration of operation was significantly longer in the laparoscopic group (Table 3).

**Table 3:** Perioperative and 1-year follow-up outcome.

	**Procedure**			
	**Open**	**Laparoscopic**	***p***	**All patients**
	***n* (%)**	***n* (%)**		***n* (%)**
Intraop. complications	1 (0.7)	1 (1.4)	0.612	2 (0.9)
General complications	5 (3.6)	1 (1.4)	0.384	6 (2.8)
Postop. complications	9 (6.5)	5 (7.2)	0.834	15 (7.2)
Bleeding	2 (1.4)	1 (1.4)	0.995	3 (2.8)
Seroma	5 (3.6)	2 (2.9)	0.793	7 (3.3)
Wound healing disorder	4 (2.9)	1 (1.4)	0.527	5 (2.4)
Infection	1 (0.7)	1 (1.4)	0.612	2 (0.9)
Reoperation	3 (2.2)	2 (2.9)	0.743	5 (2.4)
Duration of OP (mean in min)	48.7 [47.1 – 50.3]	56.1 [54.6 – 57.6]	0.025	
Time to discharge (mean in days)	3.2 [1.2 – 5.2]	3.4 [2.0 – 4.9]	0.341	
Recurrence on 1-year follow-up	3 (2.2)	5 (7.2)	0.072	8 (3.8)
Pain requiring treatment on 1-year follow-up	5 (3.6)	4 (5.8)	0.463	9 (4.3)
Seroma on 1-year follow-up	7 (5.0)	4 (5.8)	0.817	11 (5.2)
Infection on 1-year follow-up	4 (2.9)	1 (1.4)	0.527	5 (2.4)

The recurrence rate after 1 year follow-up was higher in the laparoscopic group 7.2 vs. 2.2%; *p* = 0.072 (but did not reach statistical significance). In the 1 year follow-up evaluation of pain at rest, pain on exertion and pain requiring treatment no significant differences were reported.

The inquiry at 1 year follow-up for secondary hemorrhage and infection revealed no differences between the groups (Table 3).

To identify possible differences in the patient populations with and without follow-up the relative frequencies have been calculated and are presented in Tables 4, 5. Certain differences in the relative frequencies have to be expected due to the relative small patient populations.

**Table 4:** Standardized differences of the categorical parameters between patient collectives with and without follow-up.

**Follow-up**			
	**Yes**	**No**	**Stand. diff**.
	***n*** **(%)**	***n*** **(%)**	
Male	182 (87.50)	105 (79.55)	0.216
Underweight	1 (0.48)	2 (1.52)	0.104
Normal weight	53 (25.48)	33 (25.00)	0.011
Overweight	78 (37.50)	57 (43.18)	0.116
Obesity/morbid	75 (36.06)	40 (30.30)	0.122
Laparoscopic surgery	69 (33.17)	45 (34.09)	0.019
ASA score I	1 (0.48)	2 (1.52)	0.104
ASA score II	58 (27.88)	29 (21.97)	0.137
ASA score III-IV	149 (71.63)	101 (76.52)	0.112
Elective	207 (99.52)	126 (95.45)	0.262
Recurrent operation	12 (5.77)	12 (9.09)	0.127
Defect suture	84 (40.38)	49 (37.12)	0.067
Defect size I (<4 cm)	102 (49.04)	58 (43.94)	0.102
Defect size II (4–10 cm)	102 (49.04)	69 (52.27)	0.065
Defect size III (>10 cm)	4 (1.92)	5 (3.79)	0.112
Preoperative pain	105 (50.48)	88 (66.67)	0.333
No preoperative pain	84 (40.38)	37 (28.03)	0.263
Unknown preoperative pain	19 (9.13)	7 (5.30)	0.148
Drainage	84 (40.38)	63 (47.73)	0.148
Risk factors - total	152 (73.08)	98 (74.24)	0.026
Intraoperative complications - total	2 (0.96)	1 (0.76)	0.022
General complications - total	6 (2.88)	7 (5.30)	0.122
Postoperative complications - total	14 (6.73)	15 (11.36)	0.162
Complication-related reoperations	5 (2.4)	8 (6.06)	0.182

**Table 5:** Standardized differences of the continuous parameters between patient collectives with and without follow-up.

**Follow-up**			
	**Yes**	**No**	**Stand. diff**.
	**Mean ± SD**	**Mean ± SD**	
Age (years)	69.2 ± 9.6	67.9 ± 11.1	0.127
Defect size length (cm)	4.1 ± 2.6	4.4 ± 2.6	0.115
Defect size width (cm)	3.5 ± 1.8	4.2 ± 0.6	0.336
Mesh size (cm^2^)^*^	4.9 ± 0.8	5.1 ± 0.8	0.197
Duration of operation (min)^*^	3.9 ± 0.4	4.0 ± 0.4	0.163
Time to discharge (days)^*^	1.2 ± 0.6	1.3 ± 0.7	0.124

* *Log transformed data*.

## Discussion

The repair of subxiphoidal incisional hernia following median sternotomy is technically demanding. Data are available from retrospective reports with only limited number of patients (2, 3, 5). Recently a single center study on the comparison of laparoscopic vs. open repair of subxiphoidal hernia was published (16).

This Herniamed Registry Study describes the largest cohort of patients with subxiphoidal hernia so far and delivers data on the laparoscopic and open surgical repair. However, the group of open surgical repair consists of different techniques (sublay, IPOM, onlay, component separation). In 14 cases midline approximation and suture repair was performed. This fact is astonishing as high recurrence rates up to 80% were reported for conventional mesh-free repair (4, 5).

In the group of laparoscopic repair different type of mesh fixation was used (absorbable tacks, non-absorbable tacks, suture).

According to the character of a registry-study current operation techniques are represented rather than one standardized operation procedure.

This inhomogeneity of the operation technique is the biggest limitation of the study.

Concerning predisposing factors male sex, obesity and multiple medical diagnosis (ASA > III) were predominant in patients of this study. Male sex and obesity had been identified as risk factors before (1, 4). Kim et al. however reported the opposite finding of female sex at risk for subxiphoidal hernia (17). It has to be considered that in the latter study a majority of female patients were in follow-up after coronary bypass.

An advantage was announced for laparoscopic repair of subxiphoidal hernia regarding duration of the operation and postoperative hospital stay (6, 16). For both no benefit for laparoscopic repair was detected in this study. Duration of surgery and hospital stay are considerably shorter in this study in comparison to the data published by Raakow et al. (16). The latter describes a single center study - local specifics and standards probably cause the difference.

Comparing intraoperative, general and postoperative complications no significant differences were seen between the groups. Perioperative and postoperative complication rate in this study were lower than the reported rate of 20–50% in the published series (6, 7, 16).

Assessing chronic postoperative pain there is no favor for laparoscopic or open repair. At the 1 year follow up about 3.4% of patients complained of pain at rest, about 10.6% pain on exertion, about 4.5% chronic pain requiring treatment. No data in literature are available concerning this matter.

The recurrence rate at 1 year follow-up documents a disadvantage for laparoscopic (7.2%) compared to open repair (2.2%). This finding did not reach statistical significance at the present sample size. This result, however, goes along with data published by Raakow et al. (16). It is astonishing that this effect is evident even in the heterogenous setting of open repair techniques in this registry study.

It remains to speculation whether the worse outcome of laparoscopic repair is caused by the infrequent laparoscopic closure of the hernia defect. Another explanation could be the unsolved problem of proper mesh fixation on cranial side in laparoscopic repair. The significant larger size of the used mesh prosthesis in laparoscopic technique in this study obviously did not compensate the compromising factors.

Reported recurrence rates in literature for repair using mesh prosthesis range between 10 and 30%, at follow-up time of 20–48 months (4, 6, 16). It must be assumed that the lower recurrence rates in this study correspond to the shorter follow-up time.

Limitations of the study are the variations of the operation technique especially in the open repair group and the duration of the follow-up. Furthermore, the relatively small sample size did not enable multivariable analyses.

To yield more homogenous data prospective randomized trials are desirable.

Because of the low incidence of subxiphoidal incisional hernia it will be difficult to recruit the necessary sample size even in a multi-center setting.

## Conclusion

The repair of subxiphoidal incisional hernia is safe in both laparoscopic and open technique. It is associated with a low complication rate.

The advantages reported for laparoscopic surgery—shorter operation time and duration of hospital stay, lower pain level—were not evident in this study.

Due to the lower recurrence rate evaluated for open repair in this study - preference can be given to open technique.

## Data Availability Statement

The original contributions presented in the study are included in the article/supplementary materials, further inquiries can be directed to the corresponding author/s.

## Ethics Statement

Ethical review and approval was not required for the study on human participants in accordance with the local legislation and institutional requirements. The patients/participants provided their written informed consent to participate in this study.

## Author Contributions

HA and MT analyzed the data and wrote main parts of the manuscript. FK and SG designed the study and completed the manuscript. MH provided the data and statistical tests. All authors contributed to the article and approved the submitted version.

## Conflict of Interest

FK - Grants to fund the Herniamed Registry from Johson & Johnson, Norderstedt, Karl Storz, Tuttlingen, pfm medical, Cologne, Dahlhausen, Cologne, B. Braun, Tuttlingen, Menke Med, Munich, Bard, Karlsruhe. MH was employed by the company StatConsult GmbH, Magdeburg. The remaining authors declare that the research was conducted in the absence of any commercial or financial relationships that could be construed as a potential conflict of interest.
